# Managing gender diversity and barriers to inclusion: lived experiences of women employees in a South African coal mining company

**DOI:** 10.3389/fsoc.2025.1681829

**Published:** 2025-11-07

**Authors:** Sabelo M. Baker, Thulile L. Ngonyama-Ndou, Thulani B. Skosana

**Affiliations:** 1 People Management and Development, Faculty of Management Sciences, Tshwane University of Technology, Pretoria, South Africa; 2 Department of Human Resource Management, University of South Africa, Pretoria, South Africa; 3 People Management and Development, Faculty of Management Sciences, Tshwane University of Technology, Pretoria, South Africa

**Keywords:** gender diversity, liberal feminism, mining industry, organizational culture, South Africa, women in mining, workplace inequality

## Abstract

**Introduction:**

In the context of growing global commitments to workplace diversity and inclusion, South Africa's mining sector remains a site of persistent gender inequality. Despite policy frameworks informed by liberal feminist ideals that emphasize equal rights, representation, and opportunity, women continue to experience structural and cultural barriers that hinder their advancement. This study addresses the urgent need for context-sensitive, experience-based approaches to understanding gender inequality in male-dominated industries.

**Methods:**

A qualitative, interpretive phenomenological approach was employed to explore the lived experiences of 12 purposively selected women working in various departments at a coal mining company in Mpumalanga. Semi-structured interviews were conducted to capture how these women navigate hostile workplace environments characterized by sexual harassment, gender stereotyping, unsafe working conditions, and limited access to leadership pathways. Thematic analysis was then applied using Braun and Clarke's six-step framework to identify and interpret patterns across data.

**Results:**

The findings revealed persistent structural and cultural barriers that undermine formal diversity initiatives. Participants' narratives emphasized issues of limited career advancement, biased recruitment practices, exclusionary workplace cultures, safety risks, and difficulties balancing work and family responsibilities. While liberal feminist-informed policies such as the Employment Equity Act and Women in Mining initiatives have formalized inclusion, entrenched patriarchal norms often render such reforms symbolic rather than substantive.

**Discussion:**

These findings highlight the limitations of surface-level reforms and underscore the need for deeper institutional change. Meaningful gender diversity can enhance collaboration, innovation, and workplace morale, but these benefits remain unrealised without stronger policy enforcement, inclusive organizational cultures, mentorship, gender-sensitive recruitment, safe reporting mechanisms, and targeted support for women's professional development. This research provides practical guidance for re-evaluating current diversity strategies and calls for a shift from symbolic inclusion to structural transformation within South Africa's mining industry.

## 1 Introduction

The management of gender diversity has become a priority in fostering global inclusiveness and equitable workplaces. As such, organizations increasingly recognize that diverse teams contribute to creativity, improved decision-making, and enhanced organizational performance. This is particularly magnified in traditionally male-dominated industries such as mining, where women have historically faced systemic exclusion, discrimination, and cultural barriers (Kansake and Dumakor-Dupey, 2021). In South Africa, the mining industry has long been a cornerstone of economic growth; however, it remains a challenging environment for women due to gender stereotypes, patriarchal norms, and unequal opportunities (Peltier-Huntley et al., 2022). Even though South Africa's Employment Equity Act of 1998 underscores the need to address historical imbalances by promoting gender representation, significant gaps persist within the mining sector despite progressive legislative frameworks. Consequently, women remain underrepresented in leadership roles; they experience barriers to career advancement, often working in environments that expose them to harassment, gender discrimination and workplace violence (Peltier-Huntley et al., 2022).

Despite progressive legislation such as the (Ledwaba and Nkomo 2021), women in mining remain disproportionately vulnerable. Structural and cultural barriers have long hindered full participation of women in the industry, reinforcing gendered power imbalances and limiting career progression (Botha and Cronjé, 2015). The mining sector has been known to specialize in male-centric professions due to its deep-rooted cultural practices that uphold male dominance. For instance, in South Africa, women were legally banned from working underground until the late 1900s because of the outdated perception that mining was a physically strenuous job deemed inappropriate for females (Stewart, 2020). Efforts aimed at promoting inclusivity and addressing gender inequality in South Africa's mining industry have been made, but the Minerals of Council South Africa indicates that only 15% of South Africa's mining workforce which stands at 477,000 is female, translating to roughly 72,000 women. This marks a slight improvement from earlier years, which indicates that the industry is making progress toward gender inclusivity. Thus, the Women in Mining South Africa (WiMSA) is making it easier for more women to enter and thrive in the industry. The WiMSA launched through partnerships between the Minerals Council South Africa, government, and industry stakeholders, seeks to attract, retain, and advance women by promoting mentorship, networking, and policy advocacy (Peltier-Huntley et al., 2022). The program, since its launch, has contributed to female participation to about 15% of the total workforce in the mining sector. The program has further mobilized the mentors and female role models in the sector. However, persistent issues such as unsafe working conditions, harassment, and limited promotion pathways remain, which risks rendering WiMSA symbolic rather than transformative unless paired with stronger enforcement and deeper organizational change (Kansake and Dumakor-Dupey, 2021). While policy shifts have opened job opportunities for women rooted workplace violence and harassment still make the work environment hostile (Muhayani, 2024). Moreover, the International Labor Organization points out that women are joining the mining industry in greater numbers. However, there's a lack of reliable data about their work conditions and the problems they face (Kansake and Dumakor-Dupey, 2021).

Concerningly, a recent report highlights the alarming prevalence of harassment in South Africa's mining sector which reveals that 65% of female mineworkers have experienced harassment at work, with many cases going unreported due to fear of retaliation and ineffective complaint mechanisms (Peltier-Huntley et al., 2022). Similar to this, a 2023 study by the Minerals Council South Africa revealed that almost 70% of mining women had experienced gender discrimination, from physical assault to verbal abuse (Vrankovich et al., 2025). It led to the endangering the security and mental health of female workers and these incidents discourage potential female employees from pursuing careers in the field (Mafhungo, 2022). Hence, the necessitated of targeted interventions to support women's integration and advancement in the field is also highlighted by these incidents. Organizations in the sector must be dedicated to implementing practical strategies that promote gender equality and ensure that women in mining have appropriate jobs to overcome these challenges. Therefore, scientific knowledge should be produced to develop relevant strategies.

Understanding the perception of female employees regarding management of gender diversity in a coal mine located in Mpumalanga formed the basis of this study. It focused on what women confront when attempting to foster inclusive workplace environments. The study attempted to address the research question: “What are the challenges of women in the coal mine regarding management of gender diversity?” and the study offers actionable recommendations to enhance gender diversity management within the mining sector.

## 2 Literature review

### 2.1 Gender diversity in mining

Gender diversity comprises the inclusion and representation of diverse genders within an organization to create a balanced and equitable workforce (Botha and Cronjé, 2015). Gender diversity has been acknowledged as a factor in enhancing organizational outcomes. As a result, organizations with diverse teams often report improved decision-making and innovation that include varied perspectives (Kansake and Dumakor-Dupey, 2021). However, within the mining industry, progress has been slow. Globally, women represent 16% of the mining workforce, with fewer in leadership roles (Sasikala and Sankaranarayanan, 2022). In South Africa, women comprise 15% of the 477,000-strong mining workforce, reflecting marginal improvement with the need for more focused interventions (Hilson and Maconachie, 2020). Despite legislative frameworks like the (Noge 2025), gender equity remains elusive, with gaps in policy implementation and cultural resistance viewed as significant barriers (Peltier-Huntley et al., 2022). Furthermore, global comparisons reveal that tokenistic approaches to gender equity are widespread, with a focus on headcounts rather than systemic transformation. The South African context is unique in its post-apartheid restructuring, yet similar in its institutional resistance to redistributive justice.

Research in South Africa indicates that despite growing attention to gender equity, women in the mining sector remain significantly underrepresented and face entrenched barriers to inclusion (Omotayo et al., 2020). According to the Minerals Council South Africa, women constitute only 12% of the mining workforce, with even fewer occupying leadership or technical roles (Peltier-Huntley et al., 2022). These women frequently encounter unsafe working conditions, limited access to mentorship, and inadequate facilities such as changing rooms and sanitation tailored to their needs (Anderson et al., 2021). Cultural norms and organizational structures often perpetuate masculine workplace cultures that exclude women from key decision-making processes and career advancement opportunities (Botha and Cronjé, 2015). Furthermore, challenges like compromised safety of women, lack of mentorship, and inadequate facilities tailored for women have been raise as a major concern (Kansake and Dumakor-Dupey, 2021).

Globally, similar trends persist. An international study conducted by Omotayo et al. (2020) found that women hold just 10% of leadership positions in mining, underscoring the industry-wide nature of the gender gap. In traditionally male-dominated industries gender-diverse leadership has been linked to better performance and companies with diverse executive teams are 25% more likely to outperform their peers financially (Patara, 2024). In Ghana, for instance, women in mining report being systematically excluded from operational decisions, revealing how the intersection of gender and institutional power dynamics limits their professional agency (Bahn et al., 2020). These patterns highlight the urgent need for targeted, context-specific strategies to promote gender equity in mining both in South Africa and beyond.

### 2.2 Organizational culture and policy implementation

The role of organizational culture in fostering or hindering gender diversity cannot be overlooked. Research by Shneiderman (2020) emphasized that patriarchal norms often perpetuate gender biases, particularly in male-dominated mining industries where cultural resistance frequently undermines diversity initiatives. This culture is sustained through daily micro-practices like who speaks in meetings, who mentors whom, and how performance is assessed that privilege white, male norm of professionalism (Li et al., 2022). Such informal power circuits are often invisible in HR policy but crucial in career outcomes. In South Africa, mining organizations have introduced inclusivity policies, but inconsistent implementation diminishes their effectiveness (Peltier-Huntley et al., 2022). Regrettably, diversity training programmes and anti-harassment policies are often treated as compliance exercises rather than transformative efforts. Therefore, effective policy implementation, coupled with leadership accountability and monitoring is critical to fostering inclusivity and addressing barriers women face within the mining sector.

Moreover, leadership commitment plays a pivotal role in shaping organizational cultures that either facilitate or obstruct gender diversity. Kossek et al. (2025) indicates that when senior management actively champions diversity initiatives, employees are prone to embrace inclusive practices. However, within the mining sector, leadership inertia often reinforces existing biases. diminishing well-intended policies. Without proactive leadership, diversity efforts risk being superficial, further entrenching systemic inequalities. Therefore, fostering an organizational culture that supports gender diversity requires leadership involvement, continuous diversity policy assessment, and an environment that challenges discriminatory norms.

### 2.3 Challenges to gender diversity in mining

Efforts to enhance gender diversity in mining have encountered numerous challenges like workplace culture, hiring practices, career progression barriers, workplace health and safety, work-life balance, which obstruct women's participation in the sector. The challenges are discussed below:

- Workplace Culture

The mining industry in South Africa remains steeped in masculine norms and traditions that shape its workplace culture. Historically dominated by men, mining environments often marginalize women through informal practices and implicit biases that reinforce male dominance. Women frequently report experiencing subtle exclusion from decision-making spaces, gendered jokes, and a general perception that they do not “belong” in the sector (Mahobe, 2024). This culture, characterized by hegemonic masculinities, not only undermines women's confidence but also discourages their full participation. The persistence of such cultures, despite formal diversity initiatives, reveals a disconnect between policy and practice, where symbolic inclusion fails to disrupt patriarchal norms entrenched in organizational structures (Peltier-Huntley et al., 2022).

- Hiring Practices

Gender bias in recruitment and hiring continues to limit women's entry into mining, particularly in technical and leadership roles. Although employment equity legislation in South Africa mandates non-discriminatory hiring practices, women remain significantly underrepresented in core mining functions. Stereotypes about women's physical capabilities, perceived emotional sensitivity, or unsuitability for underground work often influence hiring decisions especially in operational roles (Arditti et al., 2024). Furthermore, recruitment advertisements and selection panels are often structured in ways that unintentionally favor male candidates. The absence of gender-sensitive recruitment guidelines and insufficient outreach to qualified female candidates contribute to a slow pace of gender transformation in the sector.

- Career Progression Barriers

Women in mining frequently encounter barriers to career advancement, including a lack of mentorship, limited access to strategic networks, and opaque promotion pathways. Senior roles are often filled based on informal sponsorship or networks, from which women are routinely excluded (Galea et al., 2020). Moreover, performance evaluation systems are not always gender-neutral, with women facing greater scrutiny or having to prove themselves repeatedly. The scarcity of visible female role models in leadership further reinforces the perception that upward mobility in mining is unattainable for women. These structural and cultural impediments collectively create a “glass ceiling” that inhibits women's leadership trajectories in the sector.

- Workplace Health and Safety

Safety in mining has long been a critical concern, and for women, these concerns are magnified by gender-specific risks. Inadequate safety infrastructure such as poorly lit facilities, lack of secure changing rooms, and limited access to sanitary amenities creates an environment that is physically unsafe and psychologically unwelcoming (Basharat and Alam, 2024). Sexual harassment has also been reported by female miners in both underground and surface operations; however, this often goes unreported because of fear of punishment or a lack of trust in the reporting structures. These circumstances not only jeopardize the health of women but also make it difficult to retain them and encourage newcomers to the field.

- Work-Life Balance

Women in mining face unique complications in attaining work-life balance because of the challenging nature of mining shifts and the remote location of many operations. Unlike men, women are often expected to disguise work commitments with caregiving and family duties. The absence of flexible work schedules, family-friendly policies, or childcare assistance contributes to this pressure (Abendroth, 2022). Because of this, a lot of women either quit their jobs too soon or turn down occupation progression opportunities that require relocation or long hours. Thus, promoting work-life balance is crucial for attracting women to the field as well as ensuring their long-term success and retention. Effective tactics such as leadership commitment, cultural change, and policy enforcement are needed to remove these obstacles. By implementing best practices and adapting them to the local mining context, organizations can offer inclusive environments that support women's long-term career growth.

## 3 Theoretical framework

This study is grounded in the liberal feminist framework, which emphasizes equal rights, representation, and opportunity for women in the workplace. Liberal feminism is concerned with dismantling barriers such as discriminatory hiring practices, gender stereotypes, and exclusion from leadership pathways that reproduce inequality (Mohajan, 2022). The main tenets of liberal feminism include equal access to education and employment, gender-neutral policies, and the promotion of merit-based progression. This framework was selected because it provides a useful lens to analyse how women in mining navigate exclusionary practices, hostile workplace cultures, and structural inequalities. By linking empirical findings to liberal feminism, the study interprets the lived experiences of women not just as isolated incidents, but as reflections of broader systemic inequalities within male-dominated industries (Romano and Papastefanaki, 2020). Liberal feminist theory therefore guided the design of the study, the development of interview questions, and the interpretation of themes related to career progression, workplace culture, recruitment, and safety.

To operationalise liberal feminism in this study a multi-level relational perspective was applied to situate women lived experiences within the broader systems in which they are embedded. This perspective makes explicit how gender inequality is reproduced across different dimensions of society while aligning with the liberal feminist commitment to dismantling barriers to equality. At the micro level, women's narratives reveal everyday encounters with exclusion, harassment, and blocked promotion opportunities that affect their immediate sense of belonging at work (Mahobe, 2024). These individual experiences are inseparable from the meso level, where entrenched workplace cultures, departmental practices, and managerial attitudes reproduce patterns of inequality and limit women's professional growth (Shneiderman, 2020). Beyond the organizational sphere, the macro level includes national policy frameworks such as the Employment Equity Act and Women in Mining initiatives, which provide formal commitments to gender equity but whose weak enforcement often reduces them to symbolic gestures (Romano and Papastefanaki, 2020). Bringing these levels together illustrates how individual struggles are continually reinforced by organizational structures and national systems, thereby reproducing gendered inequalities across society (Bishu and Headley, 2020).

## 4 Research methods and design

The study was grounded in an interpretive qualitative paradigm, which assumes that reality is socially constructed, and that meaning is derived from participants' subjective experiences (Creswell and Clark, 2017). This paradigm was particularly relevant in exploring women lived realities within the mining sector, where social, cultural, and organizational contexts shape individual perceptions and meanings. This study adopted a qualitative, interpretive (also known as hermeneutic) phenomenological design. The interpretive/hermeneutic phenomenological design emphasized the interpretation and deeper meaning and understanding of lived experiences within their social and cultural contexts. Interpretive phenomenology was chosen over generic qualitative design because it provides deeper insight into how women construct meaning around their workplace experiences. While descriptive phenomenology focuses on “what” participants experience, interpretive phenomenology explores the “how” and “why” of these experiences, making it well suited for understanding systemic barriers in mining. The phenomenology design was selected as the guiding orientation because it focuses on both what participants experience and how they make meaning of those experiences within structurally unequal, male-dominated contexts (Rahi et al., 2019). Key phenomenological processes such as bracketing and reflexivity were applied to minimize researcher bias and remain attentive to participants' voices. De Bruin (2020) explains bracketing (also called epoché) as the process where a researcher intentionally sets aside or “suspends” their own prior assumptions, biases, and experiences about the research phenomenon. On the other hand, Olmos-Vega et al. (2023) state that reflexivity is the ongoing process of critically reflecting on your own role, perspective, and influence throughout the research process. Reflexivity was maintained throughout the research process, with the researcher keeping a reflexive journal to acknowledge positionality, assumptions, and potential influence on the data (Emon, 2024).

After obtaining a formal gatekeeper's letter from human resource and the ethical approval, 12 women employees were purposively selected across different departments. The recruitment process involved distributing participant information sheets and invitation letters through the company's Human Resource Department. These documents clearly explained the study's purpose, the voluntary nature of participation, and ethical considerations such as confidentiality and anonymity (Nii Laryeafio and Ogbewe, 2023). Participants who were willing to take part signed informed consent forms after the researcher provided a detailed explanation of the study and addressed any questions or concerns. Before the start of the interviews, the researcher reiterated the purpose of the study, obtained verbal confirmation of consent, and reminded participants of their right to withdraw at any stage without consequence (Schubotz, 2019). Semi-structured interviews (conducted in person) were used to elicit participants' personal accounts of navigating hostile workplace environments, including experiences of harassment, gender stereotyping, unsafe working conditions, and limited leadership opportunities. A pre-developed interview view guide with open ended questions was used to guide the interaction between the researcher and the participants. The interview guide was aligned with the objectives of the study. Where necessary, the researcher made follow-up clarity seeking questions to ensure common understanding. Each interview lasted between 20 and 40 min, was audio-recorded with permission, and later transcribed verbatim. Anonymity and confidentiality were strictly maintained, and transcripts were coded without identifiable information (Bhadke et al., 2023).

Data were analyzed using Braun and Clarke's (2006) six-phase framework for thematic analysis: familiarization with the data, generating initial codes, searching for themes, reviewing themes, defining and naming themes, and producing the report. Coding was conducted both deductively (guided by the theoretical framework) and inductively (allowing themes to emerge directly from the data). Themes such as career progression barriers and workplace culture were refined through multiple rounds of peer debriefing among authors. Trustworthiness and credibility were strengthened through triangulation maintaining an audit trail of analytic decisions and member-checking, where a summary of findings was shared with selected participants for feedback (Braun and Clarke, 2006). These strategies enhanced the reliability of the study and the validity of the findings (Emon, 2024). Although thematic analysis was the analytic tool, the phenomenological orientation of the study ensured that coding and theme development were consistently directed toward capturing and interpreting the lived experiences of women in mining.

## 5 Findings

The data revealed five themes: lack of representation and career advancement; hiring and recruitment practices; perceptions of workplace culture; work-life balance; and workplace health and safety. These themes were derived through an interpretive phenomenological analysis of data gathered from 12 in-person semi-structured interviews conducted with women employees across various departments. By emphasizing various aspects of the challenges women encounter in this male-dominated field, these themes collectively provide a comprehensive understanding of women's experiences in this field. The participants' demographic composition, included two (2) White women and three (3) women of color, contextualizes these findings. Most participants were identified as African. This racial and ethnic distribution highlights the intersectional nature of workplace inequality, as gender and race often combine to produce unique experiences in the mining industry. The researcher maintained reflexive notes during the interviews to capture non-verbal cues and contextual insights that enriched thematic interpretation, consistent with the phenomenological approach (Creswell and Clark, 2017). These themes are explored in the following sections.

### 5.1 Lack of representation and career progression

Participants expressed their dissatisfaction with the glass ceiling effect, the lack of opportunities for career advancement, the under-representation of women in career development and support, leadership opportunities, mentorship programs, and professional development. According to the findings, one of the main concerns in the mining industry is the lack of career advancement and support for women. There is a need for women to grow and advance in their careers, hence, stagnation affects the flow of work and involvement. Participants noted that women are underrepresented in leadership roles, which is disheartening and demotivating for women professionals and further said that,

“*The absence of women role models in higher positions reinforces the misconception that women are unworthy or not capable of holding such positions, we don't have those systems to guide and develop people” (Participant 2)*.

This reflects a common theme identified during interviews that participants lived experiences revealed persistent structural barriers despite having equivalent qualifications and dedication as their male counterparts. The above statement shows that most women are not provided with opportunities, career growth let alone a little bit of support to enhance themselves within the organization. Hence, it is not only of benefit to other women when women in leadership positions serve as role models, but it is also beneficial to organizations because it makes decision-making more diverse and effective.

Participant 4 felt that there are invisible barriers hinder women's progress despite their qualifications and dedication, resulting in the persistent lack of progression.

“*Despite our qualifications and dedication, there seems to be an invisible barrier that holds us back. It's like no matter what we do, the opportunities for advancement just aren't there. It's incredibly disheartening” (Participant 4)*.

In the same way, Participant 8 went into further detail about how common it is for women to be under-represented in leadership roles. Consequently, Participant 8 hinted that,

“*Women in leadership roles are not well-represented or visible.” This the idea that women are not respected or qualified to occupy influential and powerful roles within the company” (Participant 8)*.

The interpretive analysis highlighted that this perception was not isolated but consistent across departments, suggesting systemic institutional patterns rather than individual experiences. These accounts suggest that systemic limitations continue to inhibit women's professional trajectories, contributing to stagnation, frustration, and a sense of exclusion from key organizational spaces.

### 5.2 Perceptions of workplace culture

Generally, male-dominated workplace cultures are perceived as being exclusive, non-inclusive, and primarily catering to male employees. According to participant 1, “*the culture here is strongly male-dominated, and women do not feel included”*. This observation reflects patriarchal norms and structures within the workplace contributing to a lack of diversity, representation, and inclusivity, especially among women. The researcher's reflexive journal entries noted that several participants expressed visible emotional discomfort when describing workplace exclusion, reinforcing the embodied nature of these lived experiences. Cultures dominated by men are often shaped by patriarchy, which concentrates power and decision-making in the hands of men. Participant 8 went into more detail saying,

“*Navigating the male-dominated culture can sometimes make you feel alone and out of place, which makes it hard to build strong work relationships.” (Participant 8)*.

Building opportunities to ensure gender equality and help employees advance in their careers requires recognizing and overhauling male-dominated workplace norms. Participant 7 pointed out that a lack of female leaders worsens feelings of being left out:

“*There are still not many women in leadership even though they are qualified and trained. This low representation highlights ongoing barriers that stop women from climbing the professional ladder. It shows the need to make structural changes to push for equality in leadership” (Participant 7)*.

The phenomenological interpretation underscored how workplace culture is experienced not just as a social structure but as an emotional reality that influences belonging and professional identity. Signs of a non-inclusive work environment persist. Recurring patterns of exclusion, rigidity in workplace culture, and marginalization make this apparent.

### 5.3 Recruitment and hiring practices

Recruitment and hiring strategies aim to prevent issues like nepotism, favoritism political meddling, and corruption. These issues reduce the quality of employees brought into and retained within an organization. Participants perceived careful recruitment and hiring practices as follows:

“*As a woman, I feel like I have little chance of getting hired or promoted despite my qualifications. Regardless of our credentials, it seems like the industry still views men as more capable” (Participant 6)*.

“*The company is making an effort to hire more women, but it's difficult to see any real progress because of the biases we encounter. Without dealing with the underlying issue, it seems like we're only meeting quotas” (Participant 8)*.

These reflections align with the broader theme of gendered institutional practices uncovered through thematic coding. Participants commonly emphasized that recruitment systems were perceived as tokenistic rather than transformative, echoing the systemic biases described in prior research on gendered labor segmentation in mining. The results show that gender diversity can improve an organization's performance by increasing the pool of talent and encouraging new ideas when it is properly incorporated into hiring and recruitment processes.

### 5.4 Workplace health and safety

Women are often not safe in the mining industry, especially in areas where men work. Many participants view sexual harassment as an issue since they have dealt with verbal or physical abuse at work. This treatment creates an unfriendly work environment and harms both their wellbeing and happiness with their jobs.

“*Sexual harassment causes serious problems in the mines. It goes beyond just dealing with unwanted advances. Workers often feel unsafe and at risk, which makes concentrating on their job difficult. The lack of clear ways to report issues or get assistance adds to the struggle” (Participant 6)*.

*We don't feel safe there because the facilities aren't made with women in mind. Women's sense of being undervalued and unsafe at work is made worse by a lack of appropriate personal protective equipment and sanitation that meets our needs (Participant 1)*.

The researcher observed that participants spoke about these issues with heightened emotion and often paused during interviews, indicating the depth of their distress. This emphasizes the value of phenomenological inquiry in capturing the lived emotional and psychological dimensions of workplace experiences. It was frequently mentioned how emotionally taxing it is to navigate dangerous situations, which emphasizes how urgent institutional reform is.

### 5.5 Work-life-balance

Participants identified the demanding nature of the mining industry, long hours, and remote locations as major challenges when it came to juggling work obligations with those of family and providing care. Many complained that their difficulties were made worse by the absence of helpful workplace policies, such as flexible work schedules and childcare assistance.

*Balancing work and home life is practically impossible due to the lengthy shifts. Working mothers receive little consideration into account, and “we are expected to function as though we have no obligations outside of work” (Participant 3)*.

Another participant noted,

“*Women are expected to take care of the home, but we are not supported at work. Career advancement is made even more difficult by the directive to figure it out” (Participant 7)*.

The analysis revealed that these experiences were shared across participants irrespective of department, suggesting systemic organizational neglect of gender-sensitive work policies. The interpretive lens highlighted that balancing multiple social roles produced feelings of guilt, exhaustion, and diminished self-worth key emotional dimensions of women lived experiences within mining contexts. One of the main causes of discontent among female employees was the absence of flexible work schedules or childcare assistance, which led to stress, burnout, and lower retention rates.

## 6 Discussion

The findings of this study reveal that women's struggles in the mining sector cannot be studies and comprehended in isolation but must be situated within interconnected systems that continually reinforce gender inequality. At the most immediate level, participants described personal experiences of exclusion, harassment, and difficulty balancing professional and domestic responsibilities. These accounts highlight how gender inequality is embodied in everyday working lives, shaping not only career outcomes but also women's sense of safety and belonging. Prior research has similarly shown that lived experiences of harassment and blocked career progression reflect entrenched patriarchal workplace norms that limit women's participation in male-dominated fields (Bishu and Headley, 2020). From a liberal feminist perspective, these experiences reflect structural barriers that hinder women's access to equal opportunities, fair treatment, and career advancement principles central to liberal feminism's advocacy for equality through reform and institutional change (Mohajan, 2022).

These personal accounts cannot be separated from the organizational environments in which they take place. Workplace cultures that normalize exclusion, departmental practices that favor men in hiring and promotion, and managerial attitudes that undervalue women's contributions form the structural context of these challenges. Even when women demonstrate competence and commitment, invisible barriers such as the “glass ceiling” continue to block advancement. This resonates with Thelma and Ngulube (2024), who argue that gendered organizational cultures reinforce women's marginalization by reproducing traditional power hierarchies. Without transformation into these middle structures of workplace organization, women's efforts to advance remain systematically undermined. Liberal feminist theory provides a useful explanatory lens here, as it highlights the need for institutional reform through gender-neutral policies, fair promotion systems, and transparent recruitment to ensure that women are not excluded from leadership and decision-making positions (Romano and Papastefanaki, 2020).

National policy frameworks, such as the Employment Equity Act and Women in Mining initiatives, add yet another dimension to these dynamics. While progressive on paper, their weak enforcement often reduces them to symbolic commitments rather than meaningful reform (Romano and Papastefanaki, 2020). Participants' reflections align with this critique, revealing that policies intended to promote inclusion often fail to penetrate entrenched workplace cultures. As a result, reforms at the national level risk becoming surface-level gestures, leaving systemic barriers intact. In alignment with liberal feminist thought, this gap between legislative intent and practical implementation demonstrates how policy reform alone is insufficient without consistent enforcement and a transformation of social attitudes toward gender equality (Bishu and Headley, 2020).

Taken together, the findings demonstrate how inequality is reproduced across interrelated layers of experience and structure. Women's struggles with harassment and exclusion are not isolated “personal” problems, but reflections of organizational cultures and systemic policy failures. Buss et al. (2019) argue that women in South Africa are still socially typecast into narrowly defined roles, and this study extends their observation by showing how typecasting shapes career pathways in the mining industry. The persistence of these biases even in the presence of national equity legislation highlights how deeply entrenched gender inequality remains within both organizational and policy systems. Liberal feminism, with its emphasis on reform within existing social structures, reinforces the importance of addressing discriminatory practices, ensuring fair representation, and creating equal access to opportunities within the mining sector.

These findings also emphasize the limitations of piecemeal interventions. Diversity initiatives that focus only on hiring quotas, for example, may improve representation on paper but fail to dismantle patriarchal workplace norms that continue to marginalize women (Ezerins et al., 2024). Similarly, training programs designed to sensitize employees to gender issues may falter if not supported by genuine organizational commitment to structural change. Without addressing the cultural foundations of exclusion, such interventions risk reproducing the very inequalities they aim to correct. Through a liberal feminist lens, these shortcomings underscore the need for sustained, systematic reform that promotes merit-based advancement, equitable recruitment, and gender-sensitive workplace practices key tenets of liberal feminist advocacy for gender justice (Mohajan, 2022).

Addressing these issues requires sustained efforts across multiple levels. At the personal level, reporting mechanisms must empower women to safely disclose harassment without fear of reprisal (Vrankovich et al., 2025). At the organizational level, leadership structures must move beyond symbolic inclusion to actively dismantle patriarchal norms, through initiatives such as inclusive leadership training, mentorship, and transparent promotion pathways (Bridges et al., 2023). At the policy level, enforcement mechanisms for equity legislation must be strengthened so that reforms translate into material improvements in workplace practice. This resonates with Batagol and Seear (2024), who argue that gender-sensitive enforcement mechanisms are critical for making inclusion policies meaningful. This multi-level approach mirrors the liberal feminist call for equality through incremental reform, legal enforcement, and the institutionalization of fairness and transparency across all societal structures.

Finally, this study highlights the importance of adopting a relational lens to understand the persistence of gender inequality. Women's narratives of blocked advancement, unsafe environments, and work-life imbalance show that discrimination is embedded within overlapping structures, from daily interactions to departmental cultures to national policies. Unless systemic barriers are addressed holistically, interventions at any single level will remain superficial. In this sense, the mining industry reflects broader global struggles for gender equality in traditionally male-dominated sectors, underscoring the urgency of multi-level strategies that challenge inequality at its root (Ford et al., 2021).

## 7 Ethical considerations

Ethical approval was obtained from the Tshwane University of Technology Ethics Committee (RECRef2023 = 12 = 0130). A formal gatekeeper's letter was secured before data collection. Participants provided informed consent after being briefed about the study's purpose, methods, risks, and benefits. Voluntary participation was assured, with the right to withdraw at any stage without penalty. Confidentiality was preserved by assigning pseudonyms and securely storing data. Data were password-protected and access limited to the research team. To safeguard reliability, principles of beneficence and non-maleficence were applied. Sensitive issues such as harassment and discrimination were handled with empathy, and participants were offered referral to workplace support services if required. Relocating this section after the discussion aligns with standard qualitative reporting structures.

## 8 Limitations

This research focuses on just one coal mine in Mpumalanga so its findings might not apply well to other mines, areas, or industries. The study involved a purposive sample of 12 participants. While this works well for detailed qualitative research, it might not show the full range of women's experiences in the mining industry. Since the study depends on self-reported interviews, it faces possible biases like personal interpretations and memory gaps. Additionally, the lack of a quantitative element limits the capacity to assess the frequency or statistical importance of the issues noted, such as exclusion from the workplace or discrimination based on gender. The study does not particularly address how intersecting factors like race, age, and socioeconomic status could worsen gender-related barriers, despite the liberal feminist framework's emphasis on institutional reforms and equal access. The study offers transferable insights into the structural and cultural dynamics that impede gender equity, which are useful for informing gender diversity policies and practices in similarly male-dominated industries, even though the findings are context-specific and cannot be statistically generalized.

## 9 Recommendations for future research

In order to compare diversity practices and obstacles, future studies on gender diversity management in the mining industry or other male-dominated sectors should broaden their focus to include numerous businesses from different industries and geographical areas. Further insights could be gained by tracking the long-term impacts of diversity initiatives, policy changes, and cultural transformations using longitudinal studies. The use of both quantitative/mixed methods (e.g., surveys, statistics) and qualitative methods would provide a more complete picture of gender inequality. In addition, to better appreciate the influence of gender diversity issues on other aspects such as race, ethnicity, disability, socioeconomic status, and age, studies on intersectionality need to be conducted. Given that organizational cultures and leadership styles can influence the success of diversity initiatives, additional studies need to be done to see how leadership commitment propels diversity.

Surveys and case studies are necessary to evaluate the current reporting process and policy enforcement gaps, as well as to identify the efficacy of workplace safety and anti-harassment policies. Analyzing sponsorship and mentoring programmes will aid in the study of factors that influence female workers' access to and participation in such initiatives, as well as their advancement within them. The financial and organizational benefits of gender diversity, such as productivity, profitability, and innovation, should be explored to strengthen the business case for inclusive workplace policies and practices. To develop support systems and networks, cultural and psychological barriers should be investigated. This includes an examination of how workplace discrimination affects mental health, job satisfaction, and employee retention. Comparative research on gender diversity legislation in various nations or industries might provide new insights into the impact of policy frameworks on workplace inclusivity. The findings may aid in the development of more successful gender diversity initiatives and strategies by organizations and employees in the mining sector as well as beyond.

## 10 Implications for policy and practice

Addressing this historical inequality and segregation will require organizations to implement structural changes, which promote equality and reform existing hierarchies in a way that will move the mining industry forward on gender equality. In a liberal feminist framework, structural changes include a commitment to equal treatment, equal access and non-discriminatory practices. Mandating mandatory, regular training on subjects such as gender bias, organizational privilege and institutional discrimination for staff particularly those in leadership roles, is a basic place to start. Such training will have to be consistent with the South African mining industry where “race”, and class solidarity, have compounded forms of gender discrimination. Developing leaders' ability to identify and mitigate these intersecting handicaps is key to creating workplace cultures that are inclusive and equitable.

Employee hiring and retention should include focused strategies to ensure representation and equity. The bias-sensitive recruitment policy: open, fair and attuned to gender, race and class in the hiring of academic staff retention efforts must also focus on creating structured pathways that actively support women as they seek to advance, and especially for women of color. In order to monitor progress and ensure accountability these pipelines should be incorporated within performance systems and supported by robust monitoring and evaluation systems. Without these, well-intentioned diversity initiatives risk being tokenistic rather than transformational.

Action for reform must also include the workplace safety and gender-based violence (GBV) issues. Due to minimal institutional action or fear of retaliation, existing grievance mechanisms usually disregard women. With the implementation of no tolerance GBV policies along with independent and anonymous reporting systems, trust and safety cultures can be fostered. Providing personal PPE for women, safe transport, and hygienic facilities designed specifically for females working in remote or high-risk zones is essential. Liberal feminism's interventions that accommodate or tend to women's needs without sidelining their engagement in non-conventional roles agrees with what has been proposed. To help women further their professional goals, sponsoring as well as mentoring should be offered in a formal structure. Formalized mentorships must link junior female employees with experienced mentors or sponsors who are willing to advocate on their behalf and aid in important networking because informal assistance often lacks the needed structure and consistency to be truly helpful. The benefits of formal mentoring in the retention, promotion and job satisfaction of women have already been documented in similar mining contexts.

Finally, stakeholder engagement and data transparency must be the driving forces behind policy reform. Disaggregated data on hiring, promotion, compensation, and workforce composition across racial and gender lines should be regularly gathered, analyzed, and published by organizations. This kind of openness encourages institutional accountability and permits focused policy interventions. Furthermore, fostering continuous communication between female employees and leadership via consultative forums can help them jointly develop solutions to structural problems. These interactions ought to be meaningful rather than ceremonial and intended to inform real-world reforms. In conclusion, attaining gender parity in the mining industry requires a multifaceted, long-term strategy based on liberal feminist ideals of access, equality, and structural change. Mining firms can start to eliminate the systemic injustices that still prevent women from fully and equally participating in the industry by implementing inclusive policies, encouraging accountability, and rearranging organizational procedures.

## 11 Conclusion

To effectively manage gender diversity in South Africa's mining industry, a multifaceted strategy that tackles workplace discrimination, cultural biases, and structural barriers is needed. Since these issues have their roots in systemic and historical injustices, it is imperative that organizations implement all-encompassing strategies that put equity and inclusion first. Important steps include updating hiring and advancement procedures, putting in place strong mentoring programs, and encouraging an accountable culture through efficient monitoring and assessment. Leaders must thus actively promote diversity by establishing inclusive behaviors, dispelling myths, and integrating diversity objectives into organizational plans. As a result, mining firms can fully reap the rewards of gender diversity, which include improved organizational performance, strong team dynamics, and increased innovation. Notably, a diverse workforce contributes to the wider transformation of the industry and society at large by fostering competitive advantage as well as demonstrating a dedication to social justice and equality. This study advances our knowledge of how gender, race, and class co-produce exclusionary dynamics in South Africa's mining industry. It is based on intersectional feminist analysis.

## Data Availability

The raw data supporting the conclusions of this article will be made available by the authors, without undue reservation.
